# Depressive Symptoms among Haramaya University Students in Ethiopia: A Cross-Sectional Study

**DOI:** 10.1155/2020/5027918

**Published:** 2020-01-31

**Authors:** Mitiku Teshome Hambisa, Andualem Derese, Tilahun Abdeta

**Affiliations:** ^1^Haramaya University, College of Health and Medical Sciences, School of Public Health, Harar, Ethiopia; ^2^Haramaya University, College of Health and Medical Sciences, School of Nursing and Midwifery, Department of Psychiatry, Harar, Ethiopia

## Abstract

**Background:**

The prevalence of mental health problems including depression is increasing in severity and number among higher institution students, and it has a lot of negative consequences like poor academic performance and committing suicide. Identifying the prevalence and associated factors of mental illness among higher institution students is important in order to administer appropriate preventions and interventions. In Ethiopia, only a few studies tried to report associated factors of depression among university students.

**Objective:**

The objective of this study was to determine the prevalence and factors associated with depressive symptoms among Haramaya University students, Ethiopia.

**Methods:**

Institution-based, cross-sectional study design was conducted among 1040 students. A standard, self-administered questionnaire was used to get data from a sample of randomly selected 1040 undergraduate university students using a multistage systematic random sampling technique. The questionnaire used was the Beck Depression Inventory (BDI) scale which is a self-report 21-item scale that is used to assess the presence of depressive symptoms. All 21 items are rated on a three-point scale (0 to 3). Each question is scored on a 0 to 3 scale, and total scores range from 0 to 63, with higher scores reflecting greater levels of depressive symptoms. The questionnaire has been well validated as a measure of depressive symptomatology with scores 1-13 indicating minimal depressive symptoms, 14-19 showing mild depressive symptoms, 20-28 showing moderate depressive symptoms, and 29-63 indicating severe depressive symptoms. Logistic regression analysis was used to identify variables independently associated with depressive symptoms after we dichotomized the depressive symptoms screening tool to “yes/no” depressive symptoms. This means students who did not report any depressive symptoms were given “no” depressive symptoms and who reported at least one (≥1) depressive symptoms were given “yes” (depressive symptoms).

**Results:**

A total of 1022 (98.3%) out of 1040 students participated in this study. The mean age of participants was 20.9 years (SD ± 2.17), and the majority of them (76.0%) were male students. Prevalence of depressive symptoms among undergraduate students was 26.8% (95% CI: 24.84, 28.76). Among those who had reported depressive symptoms: 10%, 12%, 4%, and 1% of students reported minimal, mild, moderate, and severe depressive symptoms, respectively. Multivariable logistic regression analysis in the final model revealed that being a first-year student (AOR 6.99, 95% CI: 2.31, 21.15, *p* value < 0.001), being a second-year student (AOR 6.25, 95% CI: 2.05, 19.07, *p* value < 0.001), being a second-year student (AOR 6.25, 95% CI: 2.05, 19.07, *p* value < 0.001), being a second-year student (AOR 6.25, 95% CI: 2.05, 19.07, *p* value < 0.001), being a second-year student (AOR 6.25, 95% CI: 2.05, 19.07, *p* value < 0.001), being a second-year student (AOR 6.25, 95% CI: 2.05, 19.07, *p* value < 0.001), being a second-year student (AOR 6.25, 95% CI: 2.05, 19.07, *p* value < 0.001), being a second-year student (AOR 6.25, 95% CI: 2.05, 19.07,

**Conclusions:**

The prevalence of depressive symptoms among university students in this study is high relative to the general population. Sociodemographic factors year of study and current substance use were identified as associated factors of depressive symptoms. *Recommendations*. This finding suggests the need for the provision of mental health services at the university, including screening, counseling, and effective treatment. Families need to closely follow their students' health status by having good communication with the universities, and they have to play their great role in preventing depression and providing appropriate treatment as needed. The governments and policy-makers should stand with universities by supporting and establishing matured policies which helps universities to have mental health service centers. Generally, the university and other stakeholders should consider these identified associated factors for prevention and control of mental health problems of university students.

## 1. Introduction

Mental health disorders according to the WHO are one of the leading causes of disability worldwide [1, 2]. Currently, in the general population, the prevalence of depression is increasing as the number of people living with depression increased by 18.4% between 2005 and 2015 as people living with depression was raised to 322 million in 2015 from 17 million and five hundred thousand in 2005 [3]. Mental disorders including depression are common among higher institution students due to different academic-related and environmental factors [4–7]. These mental disorders like depression can lead students to different negative consequences including committing suicide, poor academic performance, and difficulties with interpersonal relationships [8, 9]. Depression is also one of the underlined factors for dropping out of university before completing their studies [10].

The study conducted in Ethiopia using data from the Ethiopian National Health Survey in 2012 revealed that, from 4925 adults aged 18 years and older, the magnitude of depression was 9.1% (95% CI: 8.39-9.90) [11]. However, different studies indicate that the magnitude of depression was higher among university students including the following institution-based studies which reported 27.7% and 32.2%, respectively [12, 13].

A number of factors contribute to the presentation of depression during college/university. One of them is the transition from home to college places, being away from established social support including family support systems, strive to master new skills, and increased time demands [14, 15]. In Ethiopia, being a female student, current Khat use, and being a first-year student were found to be independently associated factors of depression [12, 13].

Determining the magnitude of mental illness including depression and identifying factors associated with it among higher institution students is a pillar in order to provide appropriate interventions. However, in Ethiopia, the number of literatures reported about depression and associated factors among university students is scarce. In the current study, we aimed, additional to determining the magnitude of depression symptoms, to include more independent factors than those from the available prior studies in Ethiopia on the subject matter. These factors include sociodemographic characteristics (sex, age, religion, marital status, ethnicity, and monthly pocket money), year of study, type of high school attended, place of residence before, and current substance use.

Therefore, the objective of this study was to determine the prevalence and factors associated with depressive symptoms among Haramaya University students, Ethiopia.

## 2. Materials and Methods

### 2.1. Study Area and Period

This study was conducted from April 15 to 30, 2013, among Haramaya University regular undergraduate students. The university is located 510 km away from Addis Ababa, the capital city of Ethiopia. It is one of the oldest universities next to Addis Ababa University and the center of African excellence in Ethiopia. During the time of the study, the university had three campuses (main campus, Harar campus, and Chiro campus), 12 colleges, and 55 departments with 15,183 regular students in the undergraduate study program.

### 2.2. Study Design

The institution-based quantitative cross-sectional study design was employed.

### 2.3. Source Population

All regular undergraduate students at Haramaya University were the source population for this particular study.

### 2.4. Study Population

All students randomly selected by multistage systematic random sampling from the source population were the study population. All students who were attending their education during the time of data collection period and who were randomly selected were included.

### 2.5. Sample Size Determination

The sample size (*n* = 1040) was primarily determined by taking the prevalence of suicidal ideation among Zambian university students (31.9%) [16], 95% confidence level, the marginal error of 4%, and 10% nonresponse rate. Using the single-population proportion formula, the sample size becomes 520. Since we used multistage sampling procedure, we multiplied by design effect of 2 in order to decrease the sampling variability. Then, the final sample size became 1040.

### 2.6. Sampling Procedure

**Figure 1:**
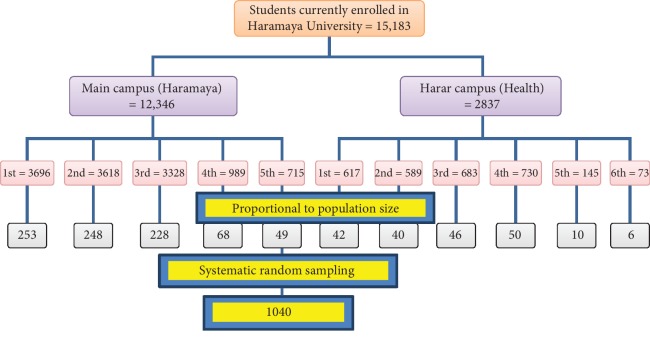
The schematic presentation of sampling procedure employed among Haramaya University students, 2013 [17].

We used a multistage sampling technique to select the study participants. In the beginning, students were categorized into two groups by their campuses (main campus and Harar campus). Then, we stratified them based on the year of study. Finally, a systematic sampling technique was employed to select students from each year of study based on their list of ID numbers in their respective batch obtained from the university registrar. Students from each year of study were allocated proportionally to their class size (Figure 1).

### 2.7. Questionnaire and Data Collection Technique

The questionnaire used was the Beck Depression Inventory (BDI) scale. The BDI is a self-report 21-item scale that is used to assess the presence of depressive symptoms. All 21 items are rated on a three-point scale (0 to 3). Each question is scored on a 0 to 3 scale, and total scores range from 0 to 63, with higher scores reflecting greater levels of depressive symptoms. The questionnaire has been well validated as a measure of depressive symptomatology with scores 0-13 indicating minimal depressive symptoms, 14-19 showing mild depressive symptoms, 20-28 showing moderate depressive symptoms, and 29-63 indicating severe depressive symptoms [18]. This standardized tool was adapted to the Ethiopian situation before it was applied for data collection. The data collectors were master degree holders who have guided the students to complete the questionnaire. The data collectors explain each question to the students to help them understand the questions well and fill their own response on the questionnaire. Facilitators were academic staffs who were familiar with the specific college who facilitated the smooth running of data collection process before and during the data collection period. The principal investigators have followed and controlled overall data collection process, trained data collectors and facilitators, and performed pretest. The data was collected using an interviewer-guided self-administered standard questionnaire which was prepared in English and then translated to local language (Amharic) which most students could understand. Then, the Amharic version was backtranslated into English by an independent translator. Then, the questionnaire was applied in the Amharic language.

### 2.8. Study Variables

The outcome variable of this study is depressive symptom, and the predictors (independent variables) are sociodemographic characteristics (sex, age, religion, marital status, ethnicity, and monthly pocket money), year of study, type of high school attended, place of residence before joining university, and current substance use.

### 2.9. Data Analysis

We used descriptive statistics to describe the characteristics of study population. Logistic regression analysis was employed to identify factors associated with the dependent variable. In order to use logistic regression, first, we dichotomized the depressive symptoms screening tool to *“yes/no” depressive symptoms*. This means students who did not report any depressive symptoms were given *“no” depressive symptoms* and who reported at least one (≥1) depressive symptoms were given *“yes”* (depressive symptoms). The odds ratio with 95% confidence interval was calculated to assess the magnitude of association and statistical significance. Those variables which were found to be significant in the bivariable analysis with a *p* value < 0.05 were retained for further multivariable logistic regression analysis to control for potential confounders and to predict independent factors associated with depressive symptoms.

### 2.10. Data Quality Control

We pretested the questionnaire on 5% of the total sample size which was 52 students of the nearby Dire Dawa University, and we made some modifications of the questionnaire based on the feedback obtained from the pretest result. Data collectors (facilitators) were trained for 2 days, and proper instruction was given before the survey. The collected data were checked for completeness and consistency before data entry.

### 2.11. Operational Definition

Beck Depression Inventory scoring: minimal depressive symptoms 1-13, mild depressive symptoms 14-19, moderate depressive symptoms 20-28, and severe depressive symptoms 29-63 [18].

### 2.12. Ethical Clearance

The project was ethically approved by the College of Health and Medical Sciences Institutional Health Research Ethics Review Committee (IHRERC) of Haramaya University. Participation was voluntary, and students were told that they can withdraw from the study at any time. Written informed consent was obtained from all participants. Confidentiality was kept, and no personal identifiers were recorded.

## 3. Results

### 3.1. Sociodemographic Characteristics of Participants

From the total of 1040 students provided with self-administered questionnaires, 1022 fully completed it (making the response rate 98.3%). Seven hundred seventy-seven (76.0%) of the respondents were males, (81.0%, *n* = 828) and the age ranged from 20 to 24 years with a mean age of 20.9 (SD ± 2.17 years). From the total participants, 41.1% (*n* = 420) were Oromo by ethnicity, 50.7% (*n* = 518) were Orthodox Christians, 90.8% (*n* = 928) were single, 34.4% (*n* = 352) were first-year students, 68.8% (*n* = 703) were originally from an urban area, 85.5% (*n* = 874) have attended a public high school, and 27.8% (*n* = 284) get a monthly pocket money of 300-499 Ethiopian birr (Table 1).

### 3.2. Magnitude and Severity of Depressive Symptoms

**Figure 2:**
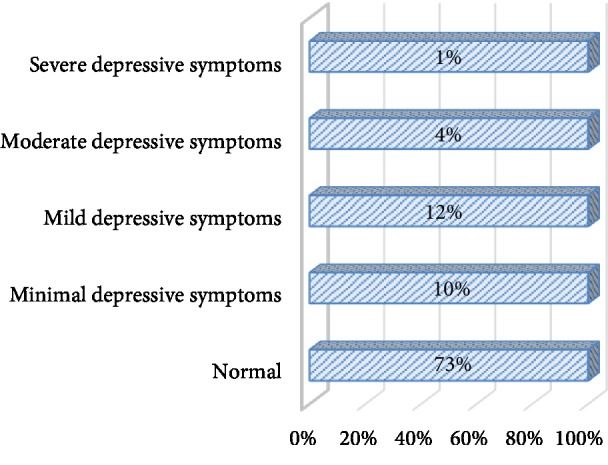
Category of depressive symptoms among the students of Haramaya University, 2013.

The result revealed that among the 1022 students of those who completed the questionnaire, using the Beck Depression Inventory scale 26.8% (95% CI; 24.84, 28.76) of them reported depressive symptoms of minimal to severe. However, the rest 73.2% did not report any depressive symptoms. Out of those who reported depressive symptoms, 10% of students reported minimal depressive symptoms, 12% mild depressive symptoms, 4% moderate depressive symptoms, and 1% severe depressive symptoms (Figure 2).

### 3.3. Factors Associated with Depressive Symptoms

We performed bivariable and multivariable logistic regression analyses in order to identify whether there is an association or not between dependent and independent variables.

### 3.4. Binary Logistic Regression Analysis

During bivariable logistic regression, variables including religion, year of study, marital status, and current chewing of Khat, drinking alcohol, smoking cigarettes, and using illicit substances have a significant association with depressive symptoms (Table 2). Then, we adjusted all these variables into the final multivariable model.

### 3.5. Multivariable Logistic Regression Analysis

In the final model, multivariable logistic regression analysis showed religion, year of study, marital status, currently drinking alcohol, smoking cigarettes, and current use of illicit substances had a statistically significant association with depressive symptoms.

The multivariable logistic regression analysis revealed that concerning their religion, traditional religion follower students were more than 3 times more likely to have depressive symptoms when compared with Protestant students (AOR 3.23, 95% CI: 1.41, 7.40). Students who were orthodox and Muslims were 2 times more likely to develop depressive symptoms as compared with being protestant, respectively (AOR 2.05, 95% CI: 1.32, 3.17) (AOR 2.02, 95% CI: 1.23, 3.33).

Being a first-year student was 7 times at a higher risk to develop depressive symptoms (AOR 6.99, 95% CI: 2.31, 21.15), and being a second-year student was more than 6 times at risk to manifest depressive symptoms (AOR 6.25, 95% CI: 2.05, 19.07) as compared with a 5^th^ year and above student. Besides, being a third-year student was more than 3 times more likely to have depressive symptoms as compared to a 5^th^ year and above student (AOR 3.85, 95% CI: 1.26, 11.78).

Being divorced/widowed was 5.91 times more likely to manifest depressive symptoms as compared to be married (AOR 5.91, 95% CI: 1.31, 26.72).

Students who had drunk alcohol at least once during the last one month were more than 2 times more likely to have depressive symptoms as compared to those who did not drink alcohol in a previous one month prior to the survey (AOR 2.53, 95% CI: 1.72, 3.72). Those who had smoked cigarettes at least once during the last one month were 1.71 times more likely to report depressive symptoms as compared to those who were not smoking cigarettes during the last one month (AOR 1.71, 95% CI: 1.02, 2.86). Those who had used illicit substances at least once during the last one month were 2.20 times more likely to develop depressive symptoms as compared to those who did not use illicit substances during the last one month (AOR 2.20, 95% CI: 1.26, 3.85).

Students who have no religion and currently chew Khat had statistically significant association with depressive symptoms in the binary logistic regression analysis. However, in the final model, they were not significant when adjusted for other variables (Table 3).

## 4. Discussion

The magnitude of depressive symptoms in our study was lower when compared to studies conducted among Malaysia university students (30.7%) [19]; Fayoum University students, Egypt (60.8%) [20]; Turkey university students (39.2%) [21]; and Ambo University students, Ethiopia (32.2%) [12]. The lower magnitude of depressive symptoms in the current study could be due to differences in the category of students included in the study. The studies by [19–21] is only among medical college students. These students are most probably at a high risk for mood disorder symptoms due to the broad content of their education both practical and theoretical parts which might stress them more and elevate the prevalence of depression and depressive symptoms [22]. Another possible reason could be due to differences in how depressive symptoms were measured. The study by [12] utilized center for epidemiological studies-depression (CES-D) scale, which asks respondents to rate how often over the past week they experienced symptoms of depression. In this method, the respondents can remember more what happened in the last one week since it is a very short period of time and this could elevate the positive response which means the prevalence of depressive symptoms.

The prevalence in this study was also higher than study findings reported among Makerere University students, Uganda (16.2%) [23]; and Australian university students (7.9%) [24]. The difference is probably due to the nature of participants. In our study, participants were only undergraduate students. However, the above study was conducted in both undergraduate and postgraduate students. Logically, postgraduate students are at a low risk to develop depression symptoms as they are more experienced with university environment. This, in turn, can decrease the prevalence of depression symptoms.

In the present study being first, second, and third-year students are more likely at risk of depression when compared to fifth-year and above students. This finding has been also demonstrated by previous studies [25]. This may show the significant role of transition associated with starting university; It suggests that the first year of university may be a particularly vulnerable period of adjustment for students because the students are separated from their families and local environments and they start a new lifestyle in the new environment. Therefore, as the students' year of study increased, the students will tolerate the university environment more and more and they will have a stable mood [26]. All the above facts show the need for interventions to decrease the prevalence of depression among students especially those who were newly commenced at a university.

In this study students, those who were divorced/widowed are more likely to have depression, as compared to those who were married. This result is in line with previous results. The death of a partner/loved one and being divorced are two of the psychosocial risk factors of depression due to lack of a partner to express their daily stressors, thereby lacking social support and social buffer [27, 28].

Also, in the current study, students who had chewed Khat, drunk alcohol and used illicit substances at least once during the last one month are more likely to experience depression. This result is in line with previous studies [27]. Even if the cause and effect are not clear in this study, this result could be due to either the fact that depressed students are more prone to substance use to relieve themselves from the depressed mood or maladaptive substance use can alter their mood to the extent of depression [28].

This study has limitation like there could be social desirability bias as the study design is cross-sectional study. There could be underreporting of the prevalence rate. Despite this limitation, the study has much strength, including the response rate, was relatively high and the sample size was large enough.

## 5. Conclusions

In this study, many university students have a high level of depressive symptoms. If not early addressed, students' depression can lead to severe complications like committing suicide and poor academic performance. Sociodemographic factors year of study and current substance use were identified as associated factors of depression. This finding suggests the need for the provision of mental health services at the university, including screening, counseling, and effective treatment. Families need to closely follow their students' health status by having good communication with the universities, and they have to play their great role in preventing depression and providing appropriate treatment as needed. The governments and policy-makers should stand with universities by supporting and establishing matured policies which helps universities to have mental health service centers. Generally, the university and other stakeholders should consider these identified associated factors for prevention and control of mental health problems of university students.

## Figures and Tables

**Table 1:** Sociodemographic characteristics of students in Haramaya University, 2013.

Sociodemographic characteristics	Frequency (*N*)	Percent (%)
Sex		
Male	777	76.0
Female	245	24.0
Age group (years)		
≤19	165	16.1
20-24	828	81.0
25-29	27	2.6
≥30	2	0.2
Ethnicity		
Oromo	420	41.1
Amara	304	29.7
Tigre	67	6.6
Wolayita	29	2.8
Harari	8	0.8
Somali	15	1.5
Guraghe	81	7.9
Others^∗^	98	9.6
Religion		
Orthodox	518	50.7
Muslim	245	24.0
Protestant	214	20.9
Catholic	6	0.6
No religion	5	0.5
Traditional religion followers	34	3.3
Year of study		
1^st^ year	352	34.4
2^nd^ year	265	25.9
3^rd^ year	249	24.4
4^th^ year	103	10.1
5^th^ year	48	4.7
6^th^ year	5	0.5
Marital status		
Single	928	90.8
Married	38	3.7
Cohabitating	41	4.0
Divorced	12	1.2
Widowed	3	0.3
Place of residence before		
Urban	703	68.8
Rural	319	31.2
Type of high school attended		
Public high school	874	85.5
Private high school	119	11.6
Missionary high school	25	2.4
Others^∗∗^	4	0.4
Monthly pocket money (Ethiopian birr)		
≤100	221	21.6
101-299	240	23.5
300-499	284	27.8
500-999	203	19.9
≥1000	74	7.2

^∗^Another ethnicity (Sidama and Hadiya). ^∗∗^Another type of high school (NGO, phase-based school).

**Table 2:** Binary logistic regression: factors associated with depressive symptoms among students of Haramaya University, 2013.

Variables	No depressive symptoms	Depressive symptoms	*p* value	COR (95% CI)
Sex				
Male	574	203	Reference	Reference
Female	174	71	0.38	1.15 [0.84-1.59]
Age group in years				
≤19	111	54	Reference	Reference
20-24	613	215	0.07	0.72 [0.5-1.03]
25-29	23	4	0.07	0.36 [0.12-1.08]
≥30	1	1	0.61	2.1 [0.13-33.5]
Ethnicity				
Oromo	312	108	Reference	Reference
Amara	222	82	0.7	1.1 [0.76-1.5]
Tigre	46	21	0.33	1.32 [0.75-2.31]
Wolayita	21	8	0.82	1.1 [0.47-2.56]
Harari	4	4	0.14	2.89 [0.71-11.75]
Somali	11	4	0.93	1.05 [0.33-3.37]
Guraghe	55	26	0.24	1.37 [0.82-2.28]
Other^∗^	77	21	0.38	0.79 [0.46-1.34]
Religion				
Orthodox	361	157	0.001	2.3 [1.53-3.47]
Muslim	181	64	0.008	1.87 [1.17-2.97]
Protestant	180	34	Reference	Reference
Catholic	5	1	0.96	1.06 [0.12-9.35]
No religion	1	4	0.007	21.17 [2.29-195.3]
Traditional religion followers	26	19	0.001	3.71 [1.71-8.04]
Year of study				
1^st^ year	240	112	0.001	5.72 [2.01-16.23]
2^nd^ year	185	80	0.002	5.3 [1.85-15.17]
3^rd^ year	187	62	0.009	4.1 [1.41-11.7]
4^th^ year	87	16	0.17	2.25 [0.71-7.12]
5^th^ year and above	49	4	Reference	Reference
Marital status				
Single	691	237	0.41	0.74 [0.37-1.49]
Married	26	12	Reference	Reference
Cohabitating	27	14	0.81	1.12 [0.44-2.88]
Divorced/widowed	4	11	0.009	5.96 [1.6-22.6]
Place of residence before				
Urban	508	195	0.32	1.17 [0.86-1.58]
Rural	240	79	Reference	Reference
Type of high school attended				
Public high school	640	234	0.72	1.1 [0.67-1.68]
Private high school	89	30	Reference	Reference
Missionary high school	17	8	0.48	1.4 [0.55-3.56]
Others^∗∗^	2	2	0.28	2.97 [0.4-21.9]
Monthly pocket money (Ethiopian birr)				
≤100	153	68	0.96	0.98 [0.56-1.74]
101-299	184	56	0.18	0.67 [0.38-1.2]
300-499	213	71	0.29	0.74 [0.42-1.3]
500-999	147	56	0.57	0.84 [0.47-1.5]
≥1000	51	23	Reference	Reference
Chewed Khat at least once during the last one month				
Yes	157	84	0.001	1.6 [1.22-2.27]
No	591	190	Reference	Reference
Drunk alcohol at least once during the last one month				
Yes	111	93	0.001	2.95 [2.14-4.1]
No	637	181	Reference	Reference
Currently smoke cigarettes				
Yes	60	50	0.001	2.56 [1.71-3.83]
No	688	224	Reference	Reference
The used illicit substance at least once during the last one month				
Yes	37	39	0.001	3.2 [1.98-5.12]
No	711	235	Reference	Reference

^∗^Another ethnicity (Sidama and Hadiya). ^∗∗^Another type of high school (NGO, phase-based school).

**Table 3:** Multivariable logistic regression: factors associated with depressive symptoms among students of Haramaya University, 2013.

Variables	No depressive symptoms	Depressive symptoms	*p* value	AOR (95% CI)
Religion				
Orthodox	361	157	0.001	2.05 [1.32-3.17]
Muslim	181	64	0.006	2.02 [1.23-3.33]
Protestant	180	34	Reference	Reference
Catholic	5	1	0.720	0.66 [0.06-6.46]
No religion	1	4	0.086	8.46 [0.74-97.14]
Traditional religion followers	26	19	0.006	3.23 [1.41-7.40]
Year of study				
1^st^ year	240	112	0.001	6.99 [2.31-21.15]
2^nd^ year	185	80	0.001	6.25 [2.05-19.07]
3^rd^ year	187	62	0.018	3.85 [1.26-11.78]
4^th^ year	87	16	0.174	2.32 [0.69-7.76]
5^th^ year and above	49	4	Reference	Reference
Marital status				
Single	691	237	0.797	0.90 [0.41-1.98]
Married	26	12	Reference	Reference
Cohabitating	27	14	0.879	1.08 [0.38-3.08]
Divorced/widowed	4	11	0.021	5.91 [1.31-26.72]
Chewed Khat at least once during the last one month				
Yes	157	84	0.770	0.94 [0.63-1.41]
No	591	190	Reference	Reference
Drunk alcohol at least once during the last one month				
Yes	111	93	0.001	2.53 [1.72-3.72]
No	637	181	Reference	Reference
Smoked cigarettes at least once during the last one month				
Yes	60	50	0.042	1.71 [1.02-2.86]
No	688	224	Reference	Reference
Used illicit substances at least once during the last one month				
Yes	37	39	0.006	2.20 [1.26-3.85]
No	711	235	Reference	Reference

## Data Availability

The datasets used and/or analyzed during the current study available from the corresponding author on reasonable request.

## Acronyms

AOR: Adjusted odds ratio
BDI: Beck Depression Inventory
CDC: Center for Disease Center
CES-D: Center for epidemiological studies-depression
COR: Crude odds ratio
GBD: Global Burden of Disease
kM: Kilometer
MSc: Master of Science
US: United States
WHO: World Health Organization.
